# Optimization in Chemical Modification of Single-Stranded siRNA Encapsulated by Neutral Cytidinyl/Cationic Lipids

**DOI:** 10.3389/fchem.2022.843181

**Published:** 2022-03-07

**Authors:** Zheng Li, Xixian Wang, Xinyang Zhou, Jie Wang, Zhu Guan, Zhenjun Yang

**Affiliations:** State Key Laboratory of Natural and Biomimetic Drugs, School of Pharmaceutical Sciences, Peking University, Beijing, China

**Keywords:** single-stranded siRNA, neutral cytidinyl lipid, chemical modification, gene silencing, biodistribution

## Abstract

Single-stranded siRNA (ss-siRNA) refers to the antisense strand of siRNA, which plays the role of gene silencing. Since single-stranded RNA is unstable, the present study employed a homemade neutral cytidinyl/cationic lipid delivery system and chemical modifications to improve its stability. The results showed that with the aid of mixed lipids, ss-siRNA could knock down 40% of target mRNA at 25 nM. With 2ʹ-F/2ʹ-OMe, phosphorothioate and 5ʹ-terminal phosphorylation, the optimized ss-siRNA could knock down 80% of target mRNA at 25 nM. Further knocking down AGO2, the ss-siRNAs could not effectively silence target mRNAs. Analysis of the biodistribution *in vivo* showed that ss-siRNA was less durable than siRNA, but spread more quickly to tissues. This study provides a safe and efficient ss-siRNA delivery and modification strategy, which lays the foundation for future works.

## Introduction

Small interfering RNA (siRNA) can inhibit the expression of target mRNA through the RNA interference (RNAi) pathway. This was first discovered by Fire and Mello et al. in *C. elegans* in 1998, for which they won the Nobel Prize in Physiology and Medicine in 2006. (Fire et al., 1998). siRNA has two strands. The first pairs the target mRNA is the antisense strand or guide strand, and the other is the sense strand or passenger strand. (Sano et al., 2008). The antisense strand can bind to Ago2 and co-factors to form an RNA-induced silencing complex (RISC). Then, RISC finds the complementary mRNA and induces cleavage. Ago2 acts as an endoribonuclease to cleave the mRNA between the 10th and 11th nucleotide, counting from 5ʹ-end of the antisense strand, hence interfering with the translation of target mRNA. (Gaynor et al., 2010).

In RNAi progress, only the antisense strand participates in Ago2 binding and mRNA cleavage, (Martinez et al., 2002), while for sense strand, it sometimes may even cause off-target toxicity due to incorrect loading. (Song et al., 2017). In 2003, Holen et al., first reported that single-stranded siRNA (ss-siRNA) could display the gene silencing effect through the same RNAi pathway as double-stranded siRNA (ds-siRNA) with much lower activity. (Holen et al., 2003). The advantages of ss-siRNA conclude lower dosage and cost due to its half molecular weight, no off-target effect caused by the sense strand incorrectly loading, etc. (Matsui et al., 2016; Pendergraff et al., 2016). However, flexible ss-siRNA is more easily to be degraded by nucleases, and it cannot activate RNAi sufficiently. (Lima et al., 2012).

Lima et al. first reported the chemical modification pattern of ss-siRNA, which contains a total of 14 phosphorothioates in dedicated sites, with alternate modifications of 2ʹ-F/2ʹ-OMe, as well as a metabolically stable 5ʹ-(*E*)-vinyl-phosphonate (5ʹ-VP) at 5ʹ-end (IC_50_ 5–20 nM). (Lima et al., 2012). The alternative 2ʹ-F/2ʹ-OMe modification can increase the stability and target binding affinity of oligonucleotides, and 5ʹ-phosphate can facilitate antisense strand binding with Ago2 protein. (Allerson et al, 2005; Prakash et al, 2015). In thus, they are the commonly used modifications in ss-siRNAs. (Yu et al., 2012; Matsui et al., 2013; Prakash et al., 2013; Liu et al., 2015; Prakash et al., 2015; Hu et al., 2017; Shen et al., 2018; Hu et al., 2019). In spite of this, some other strategies also achieve promising activity. (Haringsma et al., 2012; Chang et al., 2016). LNP is a mixed nanocarrier that contains ionizable cationic lipid, DSPE-PEG, and cholesterol. With the aid of LNP, the dosage (1–6 mg/kg i. v.) of ss-siRNA was significantly decreased. (Prakash et al., 2013).

In this study, a homemade oligonucleotides delivery system containing neutral cytidine lipid (DNCA) and cationic lipid (CLD) was used to transfect ss-siRNA. The DNCA binds with nucleic acids by hydrogen bond and π-π stacking, and the CLD through electrostatic interaction. Mixing the two lipids enriched the interaction forces between nucleic acid and lipids, thus improving the efficiency of oligonucleotides drug delivery, and reducing the use of cationic lipid, which has potential charge toxicity. Several types of oligonucleotide drugs have been successfully delivered by DNCA/CLD, with efficiency and safety *in vitro* and *in vivo*. (Ma et al., 2018; Ma et al., 2019; Zhou et al., 2020). Based on this delivery carrier, a further chemical modification was used to improve ss-siRNA’s activity. The most effective modification rules were screened out through a combination of phosphorothioate, 2ʹ-ribose substitution, and 5ʹ-phosphorylation. Further mechanism investigation was conducted by knocking down Argonaute 2 (AGO2) expression *in vitro*: accordingly, the decrease of ss-siRNA activity that reflected its action was AGO2-dependent. The ss-siRNA exhibited more rapid distribution than ds-siRNA. Overall, this study provides a safe and efficient ss-siRNA delivery and modification strategy.

## Results and Discussion

### 2ʹ-F/OMe Alternate Modification Improves the Gene Silencing Activity of Ss-siRNA

**FIGURE 1:**
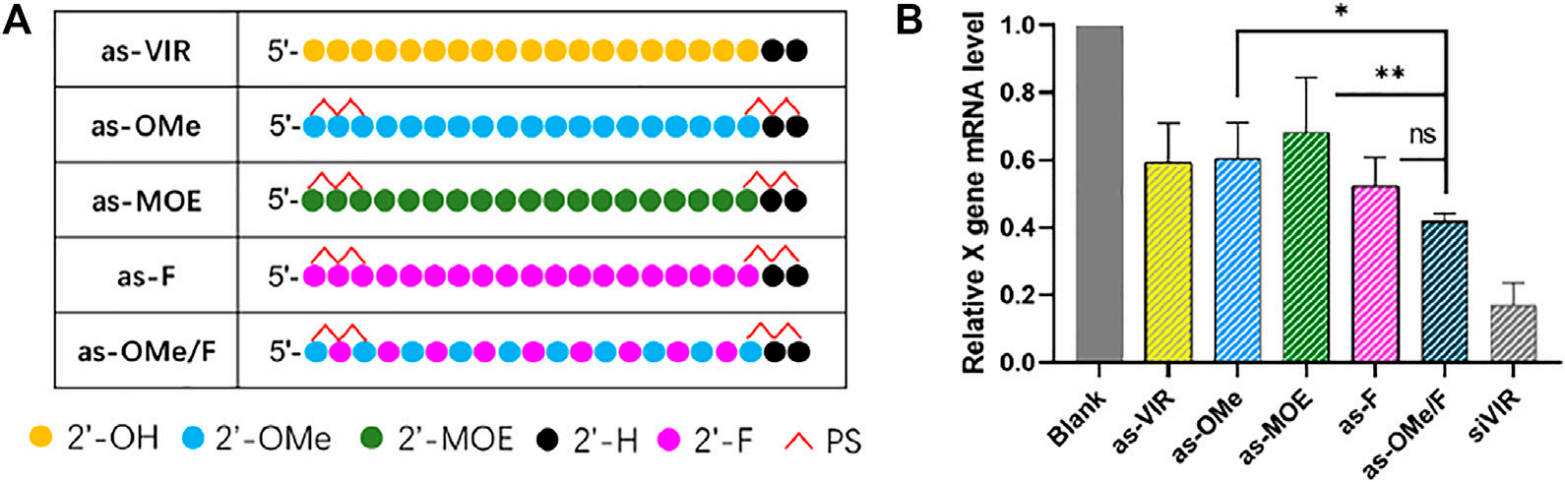
**(A)** Chemical modification of ss-siVIR **(B)** The relative expression level of X gene mRNA, measured by RT-qPCR, 24 h after transfection, 2.215 cell, at 25 nM, siRNA/DNCA/CLD = 1/21/31.5 (molar ratio). The data were shown as mean ± SD, n ≥ 3, as-OMe and as-MOE were separately compared with as-OMe/F, *p < 0.05, **p < 0.01.

Common 2ʹ-modifications of oligonucleotides include 2ʹ-F, 2ʹ-*O*-methylation (2ʹ-OMe), and 2ʹ-*O*-methoxy-ethyl (2ʹ-MOE), which replace the 2ʹ-OH of ribose to improve its nuclease resistance and target binding affinity. (Setten et al., 2019). In this study, siVIR was chosen as the prototype siRNA, which targets the X gene of the hepatitis B virus (HBV) genome and exhibits anti-HBV activity. The antisense strand of siVIR (as-VIR) was modified with different 2ʹ-modifications (Figure 1A). The ribose 2ʹ-OH of ss-siVIR fully replaced by 2ʹ-OMe, 2ʹ-MOE, or 2ʹ-F is separately called as-OMe, as-MOE, and as-F. The as-OMe/F is named by alternate modification with 2ʹ-F and 2ʹ-OMe. In addition, the ss-siRNAs were phosphorothioated on both two terminal nucleotides, which refers to the ESC strategy from Alnylam. (Nair et al., 2017). The gene silencing activity of ss-siRNA was evaluated in the HBV infection model cell line, HepG2.2.15 (abbreviated as 2.215). All siRNAs were transfected by DNCA/CLD, and the relative expression level of X gene mRNA in 2.215 after transfection was detected by RT-qPCR.

The results showed (Figure 1B) that as-VIR knocked down 40% of the target mRNA at 25 nM, which was higher than reported, reflecting the high transfection efficiency of DNCA/CLD. The activity of as-OMe was not improved from as-VIR, which was consistent with previous reports. (Haringsma et al., 2012). Although 2ʹ-OMe modification could improve the nuclease resistance of ss-siRNA, the binding affinity between ss-siRNA and target mRNA may be affected (Haringsma et al., 2012). Besides, the activity of as-MOE was lower than as-VIR and as-OMe, probably caused by its greater steric hindrance. The activity of as-F was slightly higher than as-VIR (from 40 to 50%), possibly due to the increased target binding affinity. (Haringsma et al., 2012; Hu et al., 2020).

Compared with the uniformly 2ʹ-ribose substitution (2ʹ-OMe and 2ʹ-F), the alternately 2ʹ-OMe/F is widely used in the early stage of siRNA modification, which can balance the effects of 2ʹ-OMe and 2ʹ-F on siRNA, and significantly improve its stability and target affinity. (Allerson et al., 2005; Hassler et al., 2018). In our study, as-OMe/F consistently exhibited the highest gene silencing activity (60%). Even the activity of as-OMe/F was not statistically better than as-F, and it was significantly stronger than as-OMe and as-MOE (Figure 1B). Concerning the poor stability (Kawasaki et al., 1993) of uniformly fluorine substitution, 2ʹ-OMe/F alternation is the better choice.

### Optimizing the 2ʹ-OMe and 2ʹ-F combinational modification pattern

**FIGURE 2:**
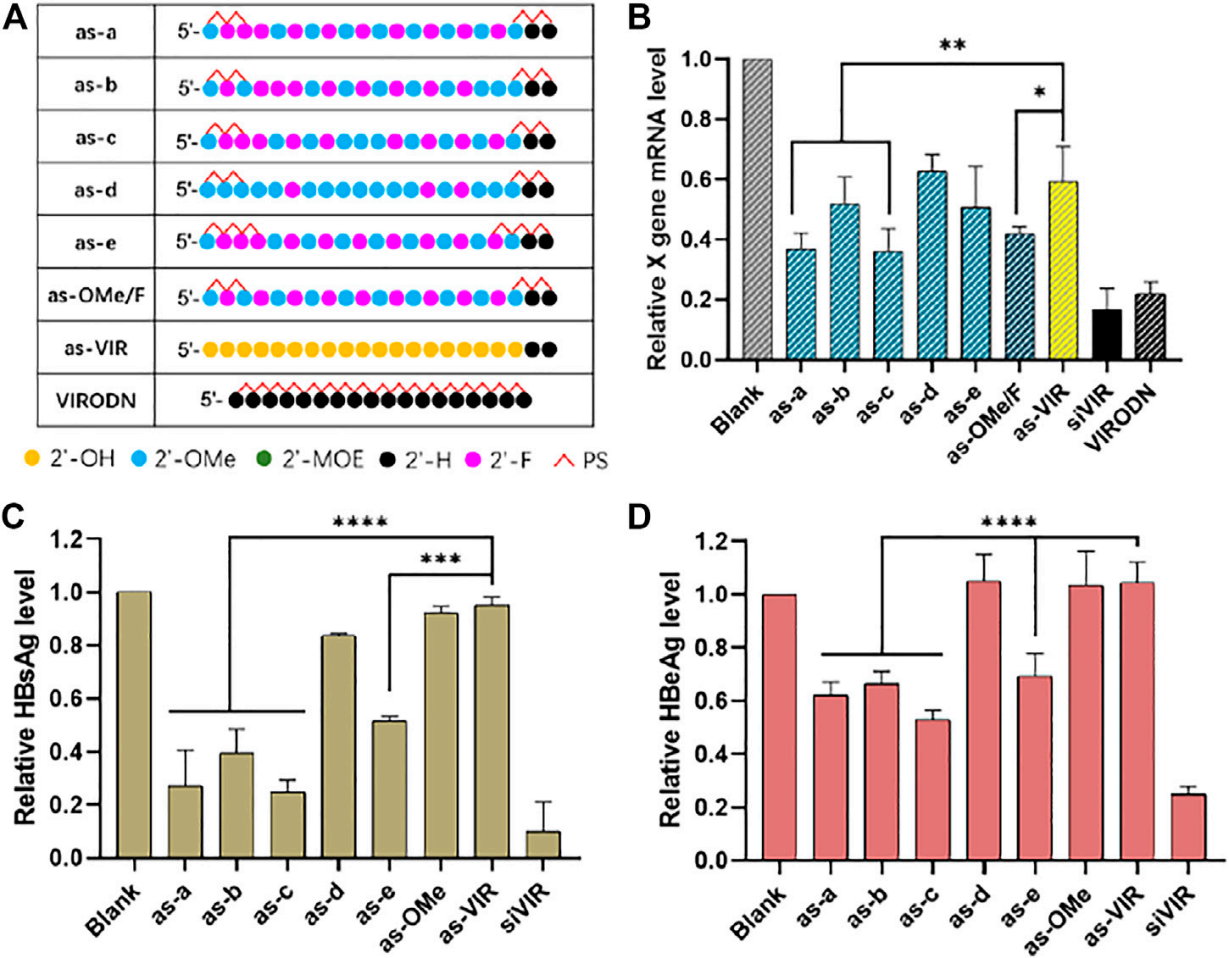
**(A)** Chemical modification of ss-siVIR; **(B)** Relative expression level of X gene mRNA, measured by RT-qPCR, 24 h after transfection; HBsAg content **(C)** and HBeAg content **(D)** in the culture medium supernatant, measured by TRIFMA, 6 days after transfection; 2.215 cell, siRNA 25 nM, siRNA/DNCA/CLD = 1/21/31.5 (molar ratio). The data were shown as mean ± SD, n ≥ 3, *p < 0.05, **p < 0.01, ***p < 0.001, ****p < 0.0001.

At present, the commonly used modification in ss-siRNA is 2ʹ-F/OMe alternation. (Lima et al., 2012). The modification strategy of siRNA has been continuously improved, proving there is a great deal of space for optimizing ss-siRNA modification. Here, concerning the ESC strategy, (Hu et al., 2020), a-e five kinds of ss-siRNA modifications were designed (Figure 2A). All the patterns were based on alternate 2ʹ-F/2ʹ-OMe with some changes in several sites. The separate activity was detected by RT-qPCR after transfection by DCNA/CLD.

As shown in Figure 2B, as-a and as-c exerted higher activity than as-OMe/F, indicating the better 2ʹ-modification arrangement, and as-c had the highest activity (65% at 25 nM). Comparing as-a with as-OMe/F, the 2ʹ-OMe at the third site of as-a was replaced with 2ʹ-F. The slightly improved activity was possibly due to the increased 2ʹ-F proportion. Comparing as-a with as-c, the difference is that 2ʹ-F at 10th site in as-a was replaced by 2ʹ-OMe in as-c. On the one hand, this proves that 2ʹ-F replacement is not better; on the other, it implies that the 10th site is probably a sensitive modification site, since it caused a stable activity difference between as-a and as-c (Figure 2B–D). However, this needs further investigation on other ss-siRNAs.

When as-a and as-e are compared, their only difference is the number of PS on both ends. As-e has 3 PSs at each end, but its activity was worse than as-a, which has 2. This suggests that the PS modification is not necessary to increase even it helps to increase nuclease resistance (Eckstein, 2014). Comparing as-b and as-OMe/F, they have the same number of 2ʹ-F/2ʹ-OMe sites but are different in the fifth and 19th sites. The reason for their different activities could be the different 2ʹ-F/2ʹ-OMe arrangement, which means that the b modification is not suitable for ss-siVIR, revealing the necessity of modification pattern optimization. As-d had the similar activity with unmodified as-VIR, presumably was due to the high proportion of 2ʹ-OMe, because the full 2ʹ-OMe modified as-OMe also had unimproved activity.

A further investigation of the antisense oligonucleotide (ASO) was conducted using VIRODN (oligodeoxynucleotide, ODN), 18nt with full phosphorothioate modification. Its sequence was the same, with 2–19 sites of the siVIR antisense strand. As shown in Figure 2B, VIRODN had potent gene silencing activity, knocking down about 80% of the target mRNA at 25 nM, which was better than ss-siRNA, presumably because VIRODN could activate the ASO mechanism more efficiently than the degree that ss-siRNA activated RISC pathway. The sequence information of ASO and ss-siRNA is almost the same, the main difference is the form of ribose, indicating the possibility of manipulating the mechanism of oligonucleotides by adjusting the form of ribose.

The silencing activity was subsequently evaluated at the post-translational level. The content of antigen (HBsAg and HBeAg) secreted in cell culture medium supernatant was detected. As in Figure 2C,D, the activity variation among all the oligonucleotides was accordant with the RT-qPCR results, but the overall activity of ss-siRNA was lower than that of the transcriptional level. The reason for this might be that ss-siRNA is less stable, so the effect cannot be sustained for a long time compared with siRNA. RT-qPCR was carried out 24 h after transfection, and the antigen detection was after 6 days. Some studies have hypothesized that ss-siRNA enters the RISC complex faster than siRNA because the double helix unwinding is unnecessary. Their results showed that the timing of maximum activity of ss-siRNA was earlier than that of siRNA and that the activity also decayed faster. (Holen et al., 2003).

In addition, to determine that the decrease of antigen content is caused by X gene silencing rather than cell death, CCK-8 testing was performed to test cell viability after the supernatant collection (Supplementary Figure S1), and the results showed that the cell survival rate had no significant difference among all the groups.

### 5ʹ-Phosphorylation Improves the Activity of Ss-siRNA

**FIGURE 3:**
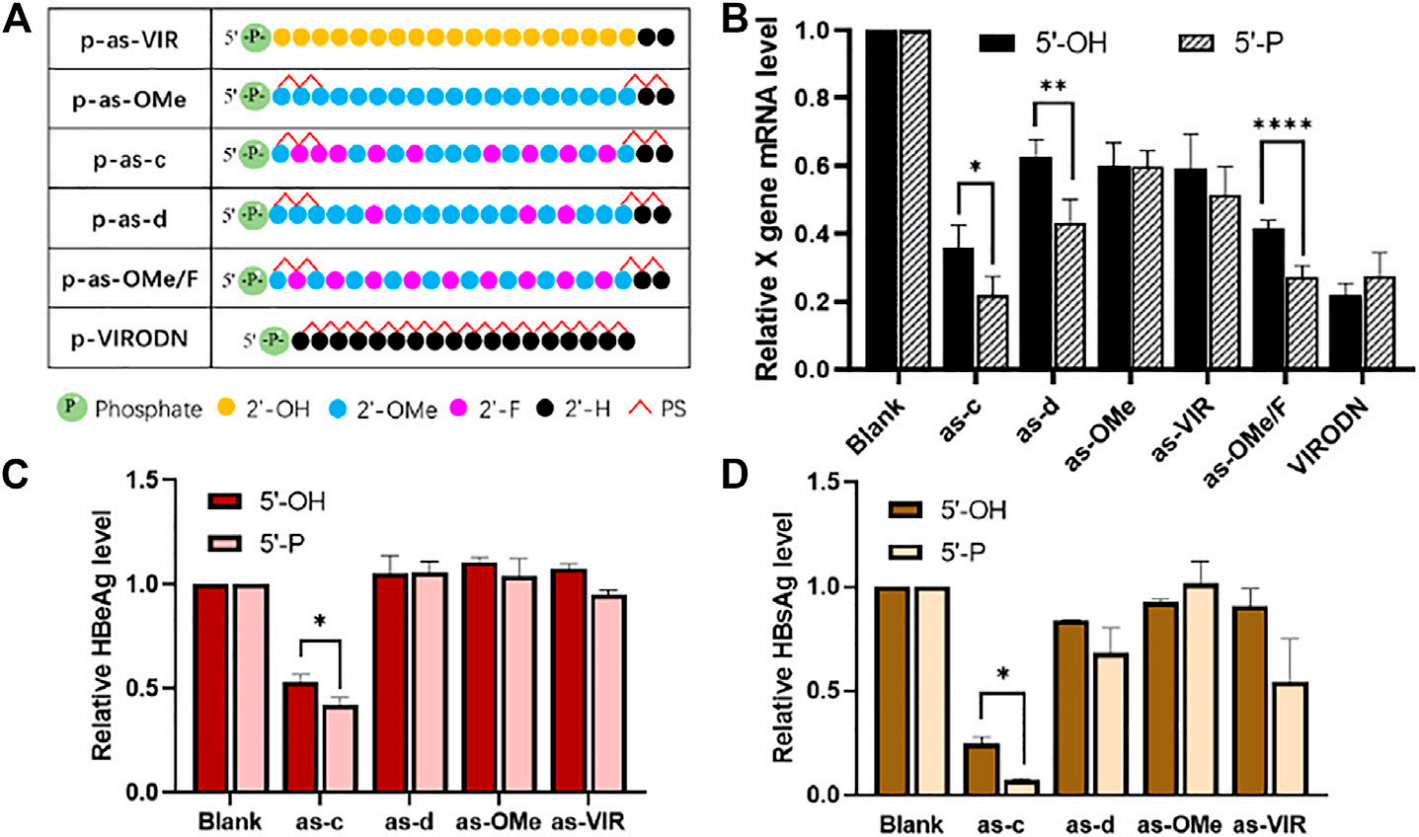
**(A)** Chemical modification of 5ʹ-P ss-siVIR; **(B)** Relative expression level of X gene mRNA, measured by RT-qPCR, 24 h after transfection; **(C)** HBeAg content in the culture medium supernatant; **(D)** HBsAg content in the cell culture supernatant; TRIFMA detection executed 6 days after transfection, 2.215 cells, at 25 nM, siRNA/DNCA/CLD = 1/21/31.5 (molar ratio). The data were shown as mean ± SD, n ≥ 3, *p < 0.05, **p < 0.01, ****p < 0.0001.

In ss-siRNA studies, people often use 5ʹ-phosphorylation modification, because 5ʹ-phosphate could help ss-siRNA to bind with Ago2, and improve its activity (Prakash et al., 2015). Therefore, in this study, a 5ʹ-phosphate group was further added to the 5ʹ-end of each ss-siRNA (Figure 3A).

As shown in Figure 3B, the activity of as-c, as-d, and as-OMe/F was significantly improved after 5ʹ-phosphorylation, and the p-as-c performed the best knockdown activity (80% at 25 nM). Figure 3C,D show the inhibition ability of ss-siRNA to HBV antigen. There is also a significant increase in as-c after 5ʹ-phosphorylation. Taken together, these results prove that 5ʹ-phosphorylation could indeed improve the activity of ss-siRNA. On the other hand, this also implies that ss-siRNA acts through the Ago2-dependant RNAi pathway because 5ʹ-phosphate is crucial for Ago2 binding. It is worth mentioning that VIRODN was not affected by 5ʹ-phosphorylation, as *p*-VIRODN had slightly lower activity (Figure 3B), which indirectly suggests that VIRODN acts through a non-RNAi pathway, most probably the ASO mechanism.

### Ss-siRNA Acts Through the RNAi Pathway

5ʹ-phosphorylation enhanced the activity of ss-siRNA (Figure 3). Additionally, the corresponding ds-siRNA showed similar variations with ss-siRNA after modifications (Supplementary Figure S2). These results indirectly indicate that ss-siRNA acts through a common RNAi pathway with siRNA. Therefore, to further understand the mechanistic action of ss-siRNA, the AGO2 knockdown experiment (Prakash et al., 2015) was performed. The activity of ss-siRNAs was evaluated after knocking down the expression of AGO2. At the same time, siNC was used as a negative control, to exclude the activity influence of competitive occupancy of siAGO2 in RNAi machinery.

**FIGURE 4:**
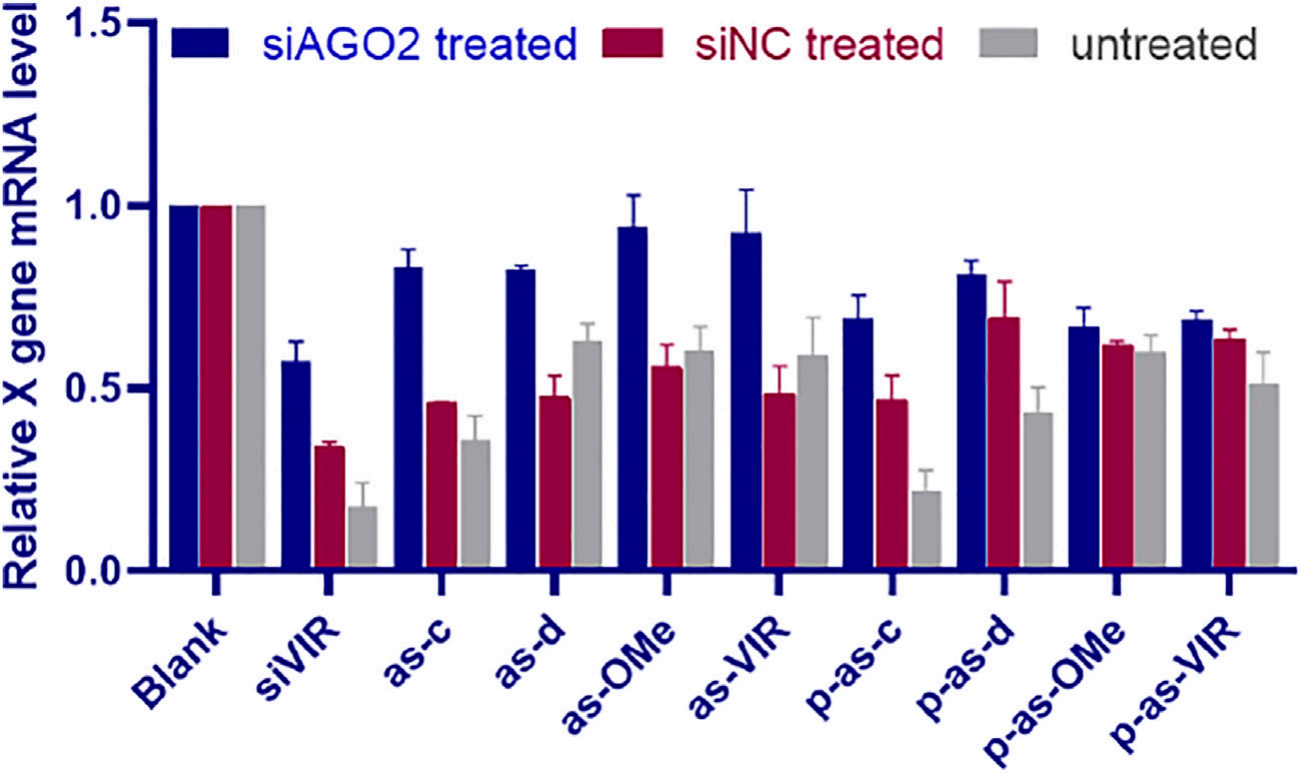
The silencing activity of ss-siRNA after AGO2 knocking down; relative expression level of X gene mRNA after siAGO2 treatment and siNC treatment, measured by RT-qPCR, 2.215 cell, siAGO2 and siNC, 25 nM, ss-siRNA, 25 nM, siRNA/DNCA/CLD = 1/21/31.5 (molar ratio). Investigate the general applicability of modification pattern in a different ss-siRNA.

**FIGURE 5:**
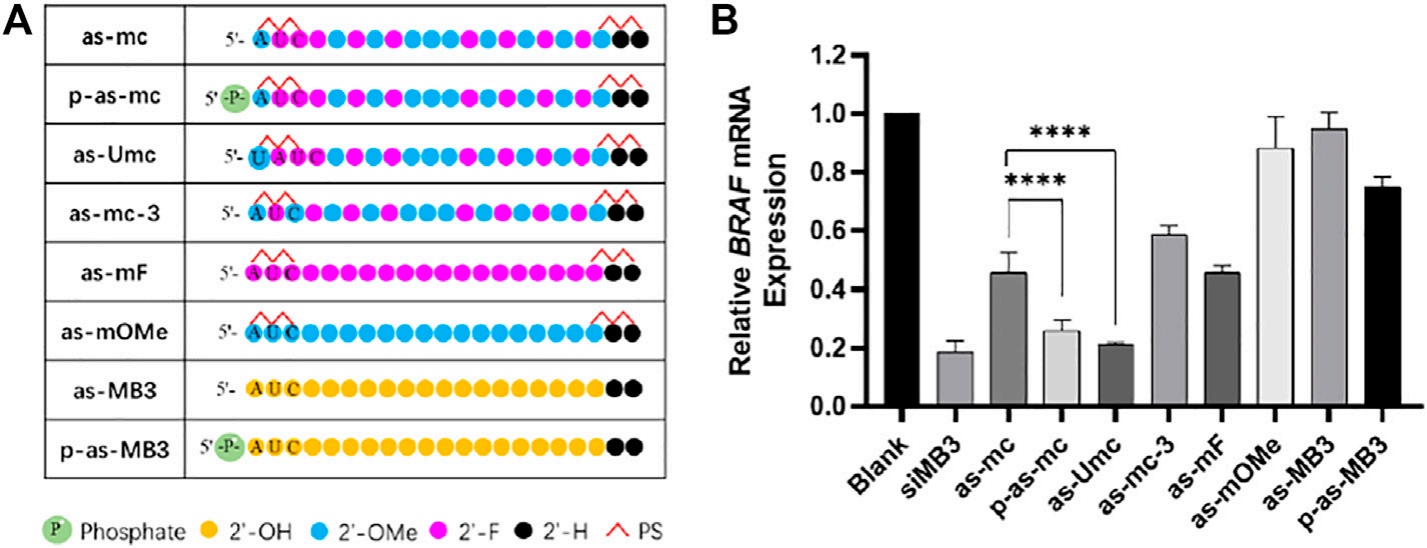
**(A)** Chemical modification of ss-siMB3; **(B)** Relative expression level of *BRAF* ^*V600E*^ mRNA, measured by RT-qPCR, 24 h after transfection, A375 cell, at 25 nM, siRNA/DNCA/CLD = 1/21/31.5 (molar ratio). The data were shown as mean ± SD, n ≥ 5, ****p < 0.0001.

As shown in Figure 4, after siAGO2 treatment, the activity of siVIR decreased compared with siNC treatment, proving that AGO2 knockdown can affect the RNAi efficiency. Meanwhile, Figure 5B shows that the activity of siNC treated is lower than the untreated group, indicating that different siRNAs will competitively use the RNAi machinery. Concerning ss-siRNA, except for p-as-d, p-as-OMe, and p-as-VIR, all the other ss-siRNAs had activity decrease after siAGO2 treatment (Figure 5A), implying that ss-siRNA most probably exerts gene silencing activity through the same RNAi pathway as siRNA. Moreover, integrated with Figure 5A, high activity strands like siVIR, as-c, and p-as-c, had a gradient descending after treating with siNC and siAGO2, compared to untreated. As a result, we inferred that for the high activity strands, their AGO2 demand is strong, so they are more susceptible to being affected by the change of AGO2 level; while for the low activity strands, the remaining level of AGO2 after knockdown or competitive binding can still meet most of their activity requirements, meaning the influence on them is slight. In addition, AGO2 knockdown was confirmed by detecting the relative expression level of AGO2 mRNA (Figure 3S).

In previous experiments, p-as-c was identified as the most effective strand, so this modification pattern was further used to another anti-tumor siRNA, siMB3, which targets *BRAF* ^*V600E*^ mRNA. BRAF mutations are widespread in melanoma, the abnormal activation of BRAF will activate the MAPK/ERK signaling pathway, which promotes the proliferation and metastasis of tumor cells. (Dong et al., 2003).

Different chemical modifications were made to the antisense strand of siMB3 (Figure 5A). As-mc and as-Umc have the same modification, but their sequences are a little different. As-Umc has a uridine (U) addition at the 5ʹ-end, which means the second to 18th sites of as-Umc are the same as the first to 17th of as-mc. The antisense strand of siVIR also has a U addition in the first site, and the second to 18th sites are complementary to the target mRNA, so the modification pattern of as-Umc is more precisely the same as as-c. As shown in Figure 5B, as-mc could knock down 54% of target mRNA, and as-Umc was 79%, which was significantly higher than as-mc, even higher than p-as-mc (74%), indicating the importance of 5ʹ-end U addition.

Meanwhile, the results showed (Figure 5B) that the activity of p-as-mc was higher than as-c, it confirmed again that 5ʹ-phosphorylation could help improve the activity of ss-siRNA. As-mF and as-mOMe are fully unitary 2ʹ-substitution ss-siMB3, the results are consistent with that of ss-siVIR, fully 2ʹ-F modification could partially improve the activity of ss-siRNA, and full 2ʹ-OMe modification is not helpful to activity improvement. As-mc-3 changed the third site modification from 2ʹ-F to 2ʹ-OMe compared to as-mc, and its activity decreased, indicating that rather than alternate 2ʹ-F/OMe, the continuous 2ʹ-F modification at the second to fourth is better. Here we found that unmodified as-MB3 almost had no potency, and the p-as-MB3 was slightly better, which are worse than as-VIR and p-as-VIR, the difference could come from the siRNA itself. Sometimes same target siRNAs with different sequences have variant activity, so it is normal for there to be some differences among different target siRNAs. Nevertheless, the modification strategy of as-Umc has potential for general applications that could enable more ss-siRNAs to perform gene silencing activity.

### Biodistribution of Chemically Modified Ss-siRNA

**FIGURE 6:**
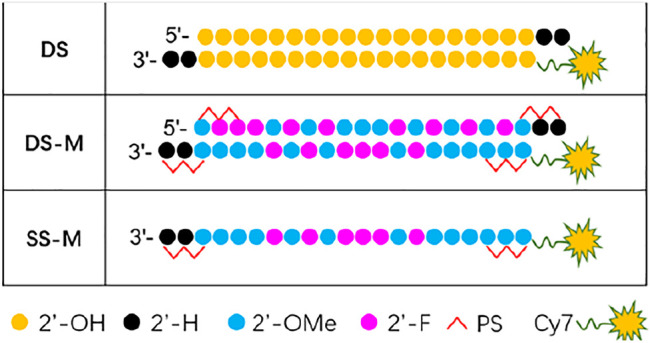
Chemical modification of ss/ds-siRNA used in biodistribution; DS: double strands; DS-M: double strands-modified; SS-M: single strand-modified.

Chemical modification is indispensable to ss-siRNA in exerting stronger activity because it helps improve stability. To further investigate the biodistribution and duration time of chemically modified ss-siRNA *in vivo*, the Cy7-labeled siRNAs (Figure 6) were administrated to BALB/c nude mice through the tail vein. They were separately unmodified siRNA (DS), modified siRNA (DS-M), and modified ss-siRNA (SS-M). All the siRNAs were encapsulated by DNCA/CLD, additionally with 0.7% PEG-DSPE to optimize the *in vivo* properties of lipoplex. (Suk et al., 2016; Zhou et al., 2020).

**FIGURE 7:**
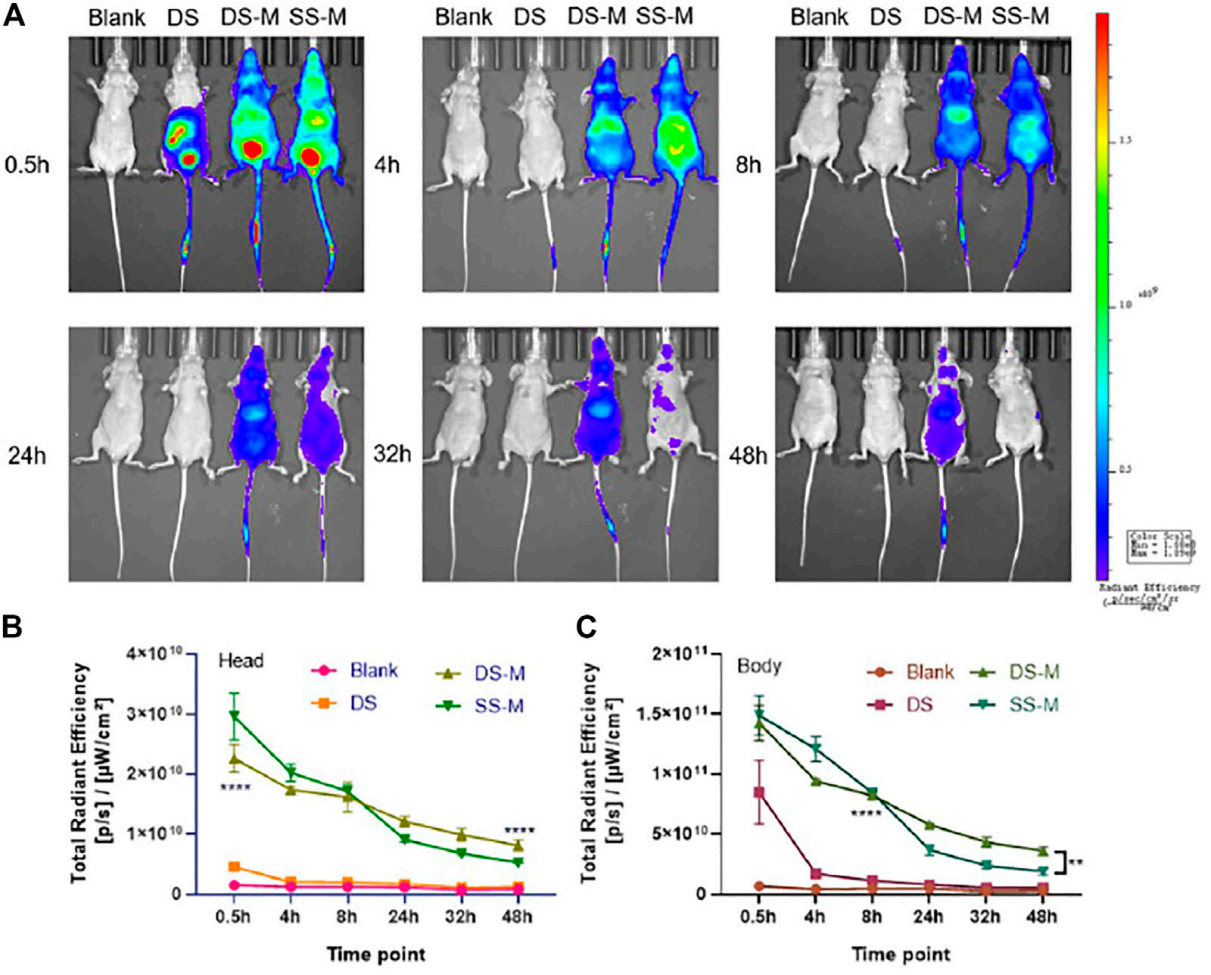
**(A)** The biodistribution of siRNA/DNCA/CLD/PEG-DSPE lipoplexes *in vivo*, BALB/c-nude mice were administrated through a tail vein; **(B)** Quantified total radiant efficiency of mouse head after treatment; **(C)** Quantified total radiant efficiency of mouse body after treatment. The quantitative results were analyzed by two-way RM ANOVA, uncoupled *p* value was compared with DS group; BLANK was solvent group; the dosage was 2 nmol per mouse (approximately DS 1.3 mg/kg, DS-M 1.4 mg/kg, SS-M 0.7 mg/kg); siRNA/DNCA/CLD = 1/21/31.5 (molar ratio), containing 0.7% PEG-DSPE molar ratio of whole CLD and DNCA. The data were shown as mean ± SD, n = 3, **p < 0.01, ****p < 0.0001.

As shown in Figure 7, the fluorescence intensity of DS-M and SS-M was always higher than that of DS, demonstrating that chemical modification well prolonged the duration time of both ss-siRNA and siRNA. Additionally, the fluorescence of the SS-M decayed faster than that of the DS-M from 24 to 48 h, which proves that the flexible ss-siRNA has poor stability than siRNA. Interestingly, at 0.5 and 4 h, a higher fluorescence of ss-siRNA was observed in the head area compared to siRNA, demonstrating that ss-siRNA distributed faster than siRNA. It might be explained by that ss-siRNAs encapsulated in lipoplexes enter cell in a different manner as that of siRNA, so it could be more easy to penetrate cell membrane, achieving faster systemic distribution. This is why the quantified result showed that the fluorescence of ss-siRNA in the head and body was higher than siRNA during 0.5–4 h (Figure 7B,C).

As for unmodified siRNA, there is no strong fluorescent signal in the head (Figure 7B), which may be due to its poor stability in blood circulation. It was more likely to accumulate in the internal organs like the liver (Figure 7C). However, after 4 h, the fluorescence decreases rapidly, reflecting its poor pharmacokinetic property.

## Conclusion

The present study has described a combinational strategy that enables ss-siRNA to exert potent gene silencing activity. We first delivered ss-siRNA with the mixed neutral cytidine lipid (DNCA) and cationic lipid (CLD), which possess enriched binding forces, achieving maintainable transfection efficiency and safety in a slight dosage (ss-siRNA/DNCA/CLD = 1/21/31.5, molar ratio). Impressively, it helped unmodified as-VIR perform 40% gene silencing activity at 25 nM.

Based on lipid delivery, an effective chemical modification pattern of ss-siRNA was further screened out. This included an additional 2ʹ-OMe modified uridine at the 5ʹ-end, then continuous 2ʹ-F modifications at 2-4 sites, and continuous 2ʹ-OMe modifications at 9–11 sites. The remaining sites are alternate 2ʹ-OMe/2ʹ-F modification, with two phosphorothiolations at both terminals. Equipped with this modification pattern, as-c and as-Umc performed desirable gene silencing activity, separately 65 and 79%.

In addition, 5ʹ-phosphorylation modification is also very important, which improves the RNAi activation ability of ss-siRNA by facilitating its AGO2 binding. P-as-c exhibited the highest target mRNA knock down potency (80%). After knocking down the expression of AGO2, the activity of ss-siRNA was accordingly decreased, implying that ss-siRNA acts through the AGO2-dependent RNAi pathway.

Biodistribution *in vivo* reflects the safety of mixed lipid delivery. Moreover, it reveals that flexible ss-siRNA is less durable than siRNA, but its transmembrane ability is improved, showing faster distribution. In summary, ss-siRNA provides a new angle in RNAi therapy. Our study has established a safe and efficient ss-siRNA delivery and modification strategy, which paves the way for future ss-siRNA works.

## Materials and Methods

### Materials

The neutral cytosin-1-yl-lipid (DNCA) and the gemini like cationic lipid (CLD) were provided by our lab. PEG-DSPE was purchased from Yuanye Bio-Technology Co., Ltd. Part of the ss-siRNA was synthesized by co-author Xixian Wang, and the other part was purchased from Sangon Biotech (Shanghai, China). The quality and bioactivity of ss-siRNA were not affected by the source.

AS-VIR: 5ʹ-UGU GAA GCG AAG UGC ACA CdTdT.

AS-MB3: 5ʹ-AUC GAG AUU UCU CUG UAG CdTdT.

### Cell Culture

The A375 cell lines were purchased from KeyGEN Biotech Co., Ltd. The hepatitis B virus model cell lines, HepAD38 and HepG2.2.15 were gifted by Fengmin Lu’s lab in Peking University Health Science Center. The medium was DMEM (M&C, China) supplemented with 10% v/v FBS (Gibco). The cells were cultured in the humidified incubator at 37°C with 5% CO_2_.

### Preparation and Transfection of Lipoplexes

siRNAs were dissolved in nuclease-free water; DNCA, CLD, and PEG-DSPE lipids were dissolved in ethanol. When preparing the lipoplexes, calculated siRNA and lipids were added in the solvent, GenOpti (M&C, China). The volume of the formulation was 10% v/v of total culture medium, that is, when the volume of culture medium was 1 ml, we seeded the cell with 900 μL medium, and prepared 100 μL formulation. The volume of ethanol was below 1% of the volume of Genopti. The mixture was then sonicated at 50°C for 10 min. As a result, the siRNA lipoplexes formulation was obtained. During transfection, the formulation was directly added to the culture medium (with 10% FBS) and incubated for 24 h.

### CCK-8 Cell Viability Measurement

The CCK-8 assay was used to detect the cell survival rate after transfection, reflecting the cytotoxicity of the formulation of lipoplexes. The cells were seeded into a 96-well plate at a certain density and grown 16–24 h to 80–90% confluence before transfection. When testing, a CCK-8 work solution was prepared, which was a mixture containing 10% CCK-8 substrate (Dojindo, Japan) and a 90% culture medium. Then the origin culture medium in a 96-well plate was removed, the 100 μL working solution was added to each well. Incubated for 1.5–2 h. The absorbance at 450 nm was measured by Tecan Spark Reader. The viability (V) was calculated by the following formula:

V = (RA-RE)/(RB-RE) × 100%

RA, RB, and RE represent the absorbance of the experimental group, the untreated group, and the blank controls, respectively.

### RT-qPCR

The RT-qPCR was used to detect the relative expression level of target mRNA, to evaluate the gene silencing activity after transfection. The cells were seeded into a 12-well plate at a certain density, grown to 80–90% confluence for 16–24 h. Then the formulation was transfected to the cells. After 24 h incubation, the cells were harvested using TRIzol (Invitrogen, United States) to extract the total RNA, stored at -80 °C. The RNA was reversed to cDNA by Reverse Transcription kit (A3500, Promega, United States), stored at -20 °C. The cDNA was then mixed with GoTaq^®^ qPCR Master Mix (A6002, Promega, United States) and primers. The qPCR progress was conducted by a real-time PCR amplifier (MX3005P). The expression level of target mRNA was calculated by the equation:

$$2^{—\left[\Delta Ct\left(A\right)—\Delta Ct\left(A\right)\right]}$$

ΔCt(A) represents the difference of the target gene threshold cycles (Ct) and housekeeping gene of each group; ΔCt(B) represents the difference of experimental groups and untreated control groups.

The primers(5ʹ-3ʹ):

X gene forward: ATG​CAA​GCT​TAT​GGC​TGC​TAG​GCT​GTA​CTG.

X gene reverse: TGC​GAA​TTC​TTA​GGC​AGA​GGT​GAA​AAA​GTT​G.

MB3 forward: TGG​TGT​GAG​GGC​TCC​AGC​TTG​T.

MB3 reverse: ATG​GGA​CCC​ACT​CCA​TCG​AGA​TTT​CT

β-actin forward: CCAACCGCGAGAAGATGA

β-actin reverse: CCA​GAG​GCG​TAC​AGG​GAT​A.

### Time-Resolved Immunofluorometric Assay

The content of antigens (HBsAg, HBeAg) produced by hepatitis B virus model cells was measured following the protocol of the Hepatitis surface/e antigen detection kit (Xinbo Biotech Co., Ltd. China). The 2.215 cells were seeded into a 96-well plate at 4×10^4^ per well, incubated for 16–24 h to grow to 80–90% confluence, then the lipoplexes were added into the culture medium. After 6 days of incubation, the supernatant was collected for antigen detection. The time-resolved immunofluorometric analyzer (ANYTEST) was used to detect the antigen content of samples.

### Knockdown of AGO2 Expression

The cells were seeded into a 12-well plate at 1×10^5^ per well. After 16–24 h incubation, the lipoplexes of siAGO2/DNCA/CLD was added to the culture medium, siAGO2 was used as a pool of four different siRNAs (Supplementary Table S1) targeting AGO2 mRNA at a total 25 nM (siAGO2-1 + siAGO2-2 + siAGO2-3 + siAGO2-4, 6.25 nM each). After 24 h incubation, the lipoplexes of ss-siRNA/DNCA/CLD were added to the culture medium and incubated for another 24 h. The cells were harvested using TRIzol (Invitrogen, United States) to extract the total RNA, stored at −80°C. RT-qPCR was used to measure the knockdown efficiency of siAGO2 and ss-siRNA.

### Animals

All animal experiments were approved by the Committee for Animal Research of Peking University (No. LA2017194). All the operations involving animals were conformed to the National Institutes of Health Guide for the Care and Use of Laboratory Animals (NIH Publications No. 8023, revised 1978). The specific pathogen free (SPF) grade female nude mice (3–4 weeks) were obtained from Wantonglihua (China) and kept in the Department of Laboratory Animal Science, Peking University Health Science Center.

### Biodistribution Assay *in vivo*

Each group contained three mice. The lipoplexes of Cy7-siRNA/DNCA/CLD/PEG-DSPE were injected through the tail vein, the dosage of siRNA was 2 nmol per mouse (approximately ss-siRNA 0.7 mg/kg, siRNA 1.4 mg/kg), the injection volume was 200 μL. The images were taken at 0.5, 4, 8, 24, 32, 48 h after injection utilizing the IVIS^®^ Spectrum *in vivo* imaging system (PerkinElmer, United States). The measurements were performed at 745 nm of excitation wavelength and 800 nm of emission wavelength. The experimental data were analyzed by Living Image^®^ 4.3.1 software.

### Statistical Analysis

All the data showed the mean of experimental values, and the error bars were standard deviations (n ≥ 3). The statistical analyses were performed by GraphPad Prism 8 software, and Student *t*-test or Welch *t* test was used for comparison between two groups. For multiple groups, the one-way ANOVA analysis of variation was used. The biodistribution quantitative data were analyzed with a two-way RM ANOVA. As appropriate, the significant differences were represented by *p*-values: n. s. *p* > 0.05; *p < 0.05; **p < 0.01; ***p < 0.001; or ****p < 0.0001.

## Data Availability

The original contributions presented in the study are included in the article/Supplementary Material, further inquiries can be directed to the corresponding author.
