# Supplementary material for: Optimization in Chemical Modification of Single-Stranded siRNA Encapsulated by Neutral Cytidinyl/Cationic Lipids
Source: Front Chem. 2022 Mar 7;10:843181. doi: 10.3389/fchem.2022.843181 (PMC8957067; doi:10.3389/fchem.2022.843181)
Supplement: Supplementary file 1 [file DataSheet1.PDF]

Supplementary Information

Table S1. The sequence of siAGO2

Name		sequence 5′-3′
siAGO2-1	AS	pUUC AGA UGG ACU UCC GUG CUU
	SS	GCA CGG AAG UCC AUC UGA AUU
siAGO2-2	AS	pUUC AGA UGG ACU UCC GUG CUU
	SS	GCA GGA CAA AGA UGU AUU AUU
siAGO2-3	AS	pUAA UAC AUC UUU GUC CUG CUU
	SS	GGG UCU GUG GUG AUA AAU AUU
siAGO2-4	AS	pUGA CAU UGG GUU CUC AUA CUU
	SS	GUA UGA GAA CCC AAU GUC AUU

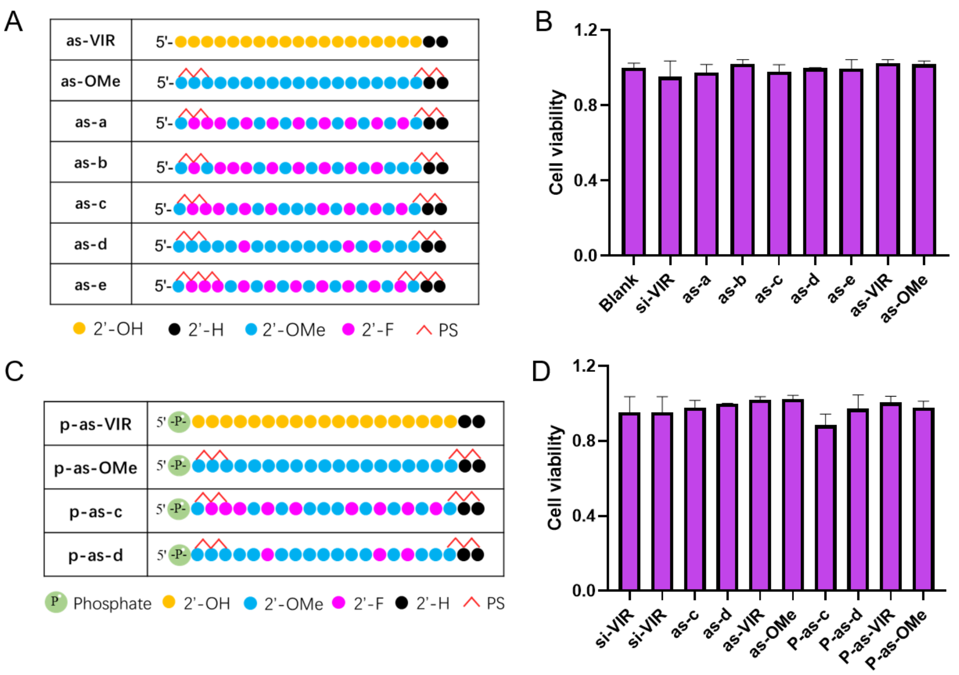

Fig. S1. The cytotoxicity of lipoplexes, measured by CCK-8 assay, 6 days after transfection, siRNA/DNCA/CLD = 1/21/31.5 (molar ratio), 2.215 cells, siRNA = 25 nM.

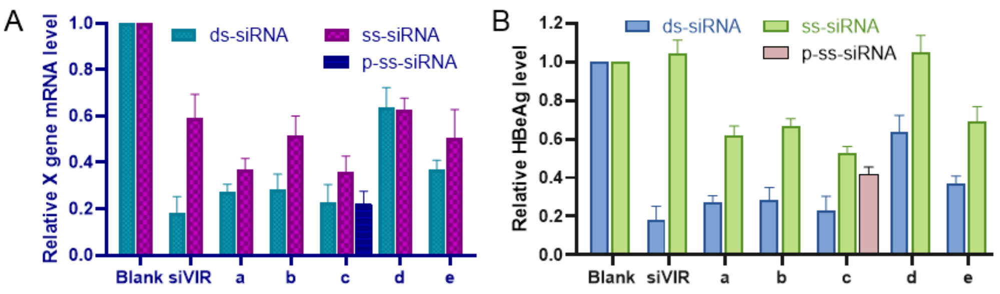

Fig. S2. Comparison of the activity of double-stranded (ds-siRNA) and single-stranded (ss-siRNA), “a-e” represents five modification patterns, “p” represents 5′-phosphorylation; A. Gene silencing activity, measured by RT-qPCR; B. Inhibition ability of HBsAg produce, antigen content in supernatant of culture medium was measured by TRIFMA, siRNA/DNCA/CLD = 1/21/31.5 (molar ratio), 2.215 cells, siRNA = 25 nM.

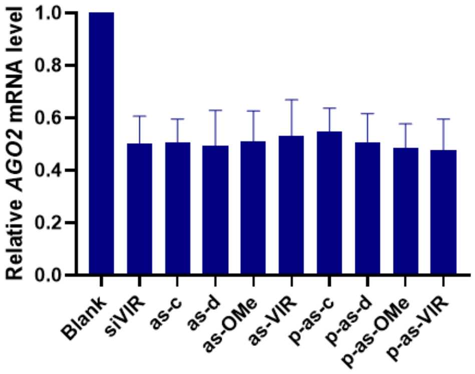

Fig. S3. The knockdown activity of siAGO2; relative *AGO2* mRNA expression level was measured by RT-qPCR, siRNA/DNCA/CLD = 1/21/31.5 (molar ratio), 2.215 cells, siRNA = 25 nM.

All of RNAs (Table S2) have been synthesized with ABI 394 DNA/RNA synthesizer with DMT-on strategy. After that, the CPG powder was collected in a 3 mL of glass bottle. RNAs were cleaved from CPG by using 2 mL aqueous ammonium hydroxide (25-28%, ω%) at 60°C for 16 h. Then the solution was collected and concentrated by vacuum evaporation.

All the single strand RNAs were purified by GILSON HPLC under the reversed-phase condition (XBridge™ OST C18 OBD™ 2.5 μm 19×50 mm Column): A: 0.05 M triethylammonium bicarbonate buffer (TEAB, pH 8); B: acetonitrile; flow rate: 4 mL/min, B: 15-40% in 25 min. The result of HPLC is shown in Fig. S4. The target fraction solution was collected and concentrated by vacuum evaporation. The residue was dissolved with 3 mL water, then add 0.6 mL Trichloroacetic acid solution (0.5 M in water), after reacting for 3 minutes at room temperature, add 0.6 mL sodium bicarbonate aqueous solution (0.5 M) and the DMT protecting group is removed from RNA. Then the RNAs were purified by GILSON HPLC with desalting column (HiPrep™ 5 mL Desalting): flow rate: 2 mL/min, mobile phase: pure water. The result of desalting is shown in Fig. S5. The target fraction solution was collected and concentrated by freeze dryer (CHRIST ALPHA 2-4 LD plus). The target product fraction was characterized by electrospray ionization mass (ESI-MS).

Table S2. The single strands sequence and Mass

Name	Sequence(5′-3′)	Calcd.	Found
as-mc	Ao*Uf*Cf GfAoGf AoUfUo UoCoUf CoUfGo UfAoGf Co*dT*dT	6830.4	6830.6
as-umc	Uo*Af*Uf CfGoAf GoAfUo UoUoCf UoCfUo GfUoAf Go*dT*dT	6831.4	6832.5
as-mc-C	Ao*Uf*Co GfAoGf AoUfUo UoCoUf CoUfGo UfAoGf Co*dT*dT	6842.5	6842.8
as-mc-F	Af*Uf*Cf GfAfGf AfUfUf UfCfUf CfUfGf UfAfGf Cf*dT*dT	6710.1	6710.5
as-mc-OMe	Ao*Uo*Co GoAoGo AoUoUo UoCoUo CoUoGo UoAoGo Co*dT*dT	6938.7	6939.3
as-VIR-F	Uf*Gf*Uf GfAfAf GfCfGf AfAfGf UfGfCf AfCfAf Cf*dT*dT	6834.2	6834.6
as-VIR-OMe	Uo*Go*Uo GoAoAo GoCoGo AoAoGo UoGoCo AoCoAo Co*dT*dT	7062.9	7064.4
as-VIR-MOE	Ue*Ge*Ue GeAeAe GeCeGe AeAeGe UeGeCe AeCeAe Ce*dT*dT	7956.0	7957.4

Af/Gf/Cf/Uf represents the nucleotide at this position is modified with 2′-F, Ao/Go/Co/Uo represents the nucleotide at this position is modified with 2′-OMe, and Ae/Ge/Ce/Ue represents the nucleotide at this position is modified with 2′-*O*-MOE, dT represents the nucleotide at this position is thymidine, and the upper right corner marked with "*" means that the position is modified by phosphorothioate.

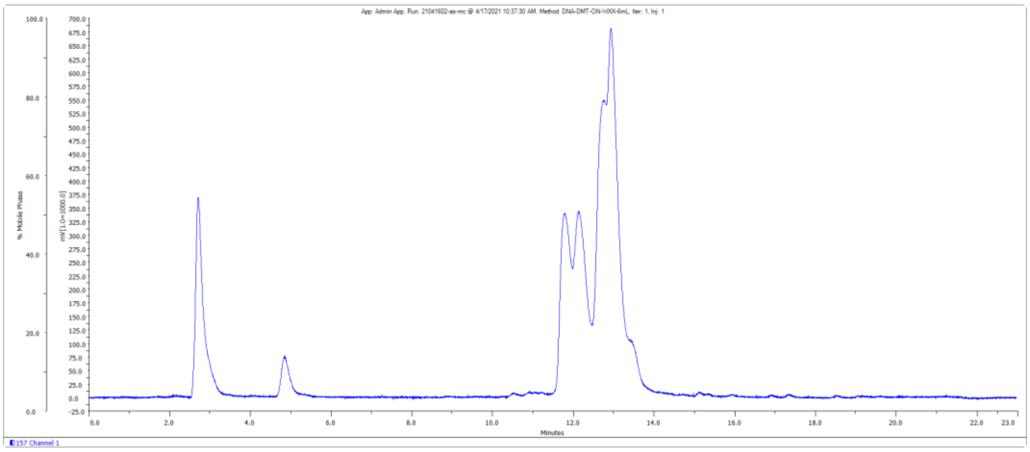

Fig. S4. The HPLC result of as-mc. The retention time of the target product is between 11.6-13.6 min.

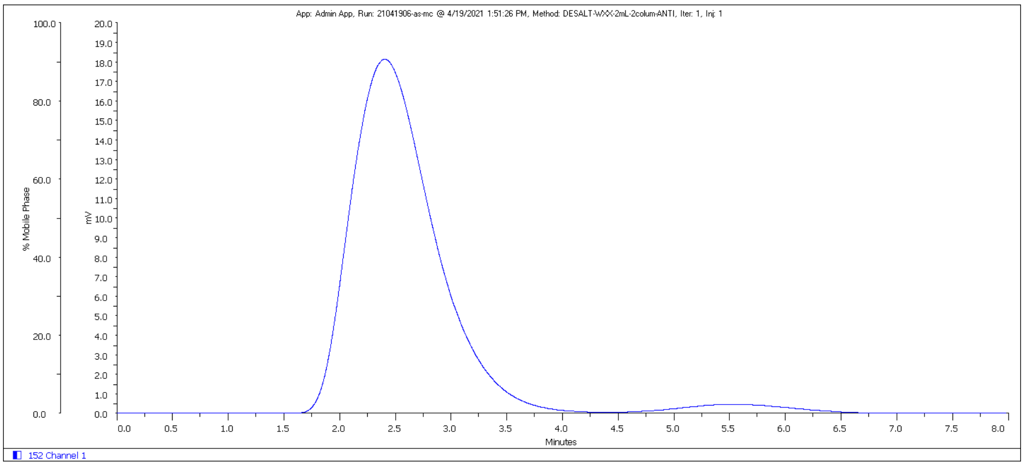

Fig. S5. The desalting result of as-mc. The retention time of the target produce is between 1.7-3.2 min.

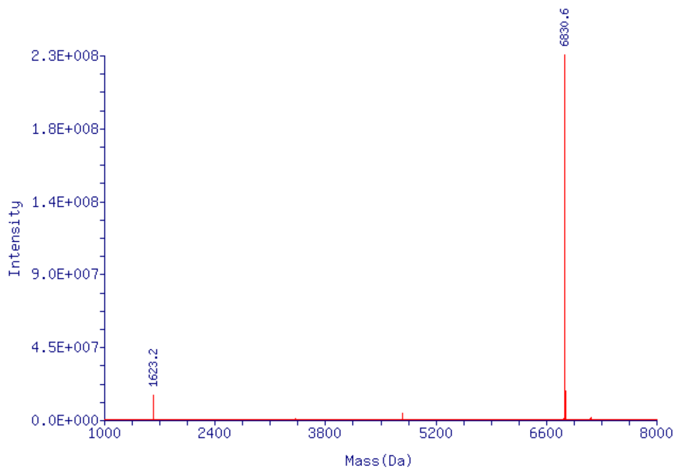

Fig. S6. ESI-TOF Mass spectra of as-mc

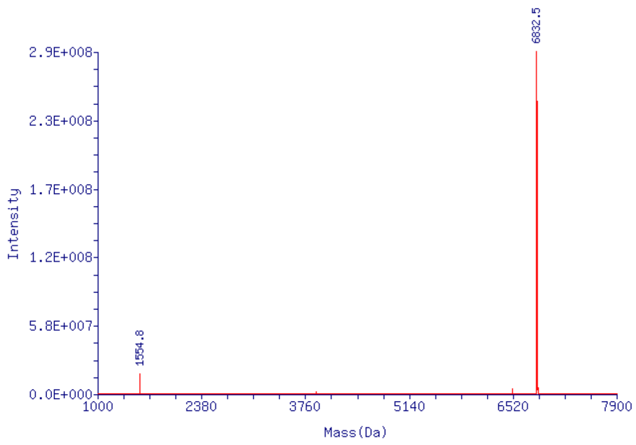

Fig. S7. ESI-TOF Mass spectra of as-umc

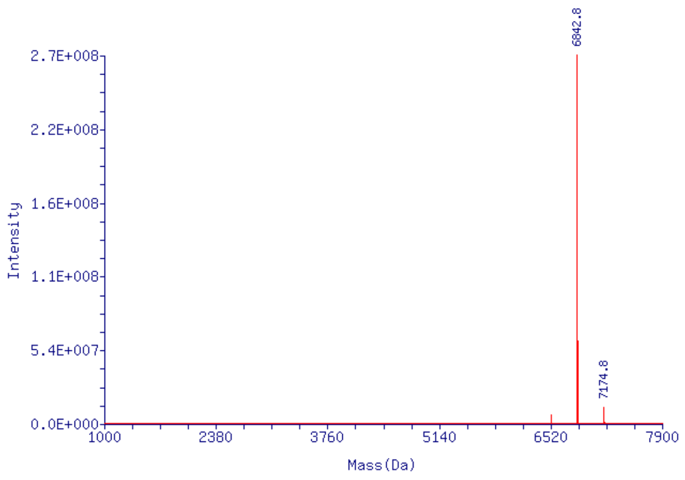

Fig. S8. ESI-TOF Mass spectra of as-mc-C

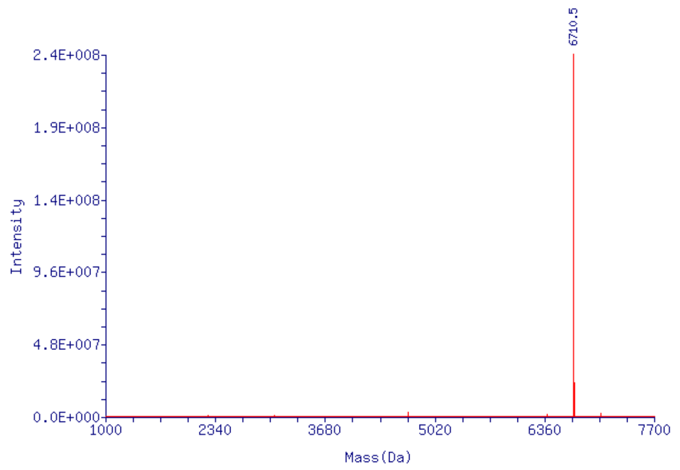

Fig. S9. ESI-TOF Mass spectra of as-mc-F

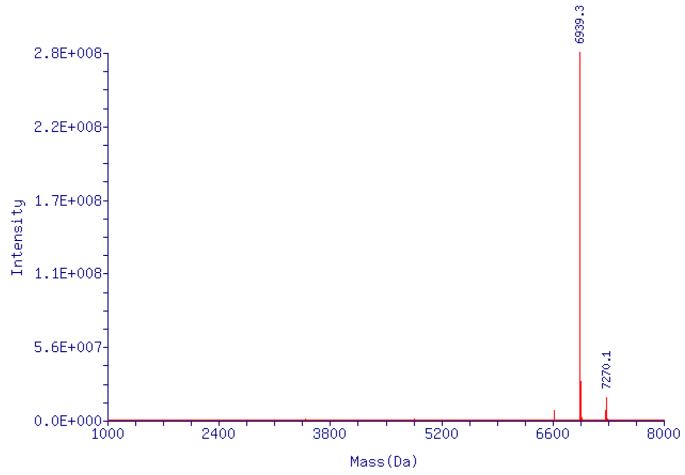

Fig. S10. ESI-TOF Mass spectra of as-mc-OMe

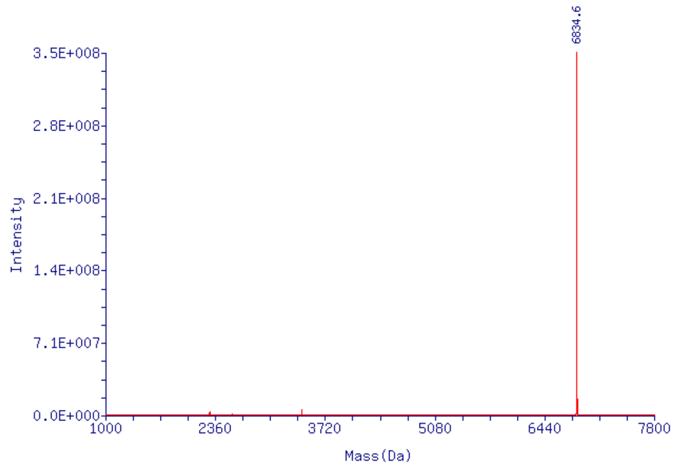

Fig. S11. ESI-TOF Mass spectra of as-VIR-F

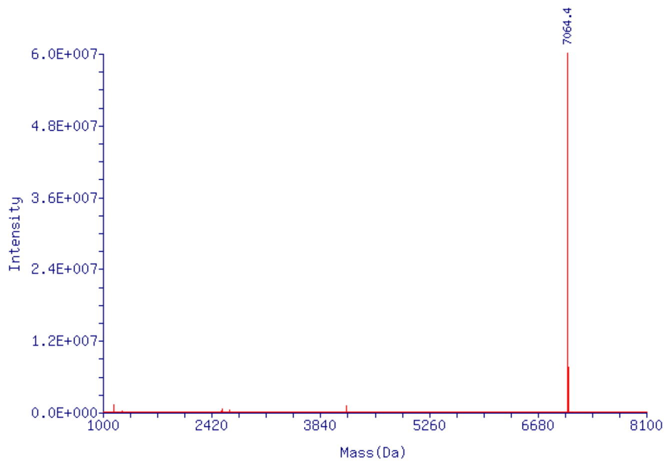

Fig. S12. ESI-TOF Mass spectra of as-VIR-OMe

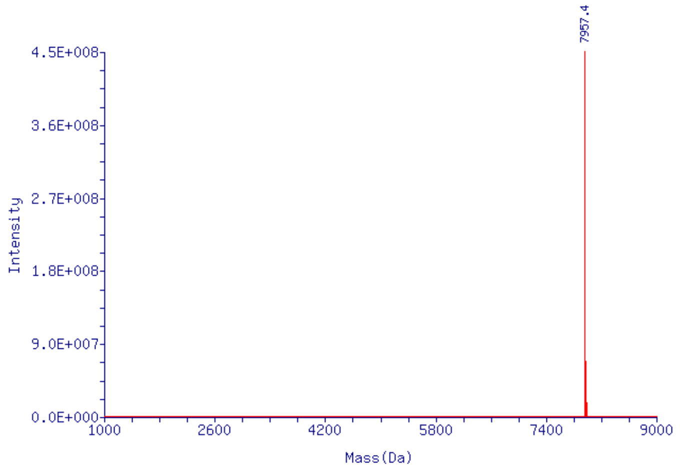

Fig. S13. ESI-TOF Mass spectra of as-VIR-MOE
