# Supplementary material for: Optimization in Chemical Modification of Single-Stranded siRNA Encapsulated by Neutral Cytidinyl/Cationic Lipids
Source: Front Chem. 2022 Mar 7;10:843181. doi: 10.3389/fchem.2022.843181 (PMC8957067; doi:10.3389/fchem.2022.843181)
Supplement: Supplementary file 2 [file Image1.pdf]

Gene Silencing and Biodistribution of 2′-Modified Single-stranded siRNA Encapsulated by Neutral Cytidinyl/Cationic Lipids

[†]Zheng Li, [†]Xixian Wang, Xinyang Zhou, Jie Wang, Zhu Guan, Zhenjun Yang[*]

State Key Laboratory of Natural and Biomimetic Drugs, School of Pharmaceutical Sciences, Peking University, Beijing 100191, China.

[†]These authors have contributed equally to this work and share first authorship. [*]yangzj@bjmu.edu.cn

Graphical abstract

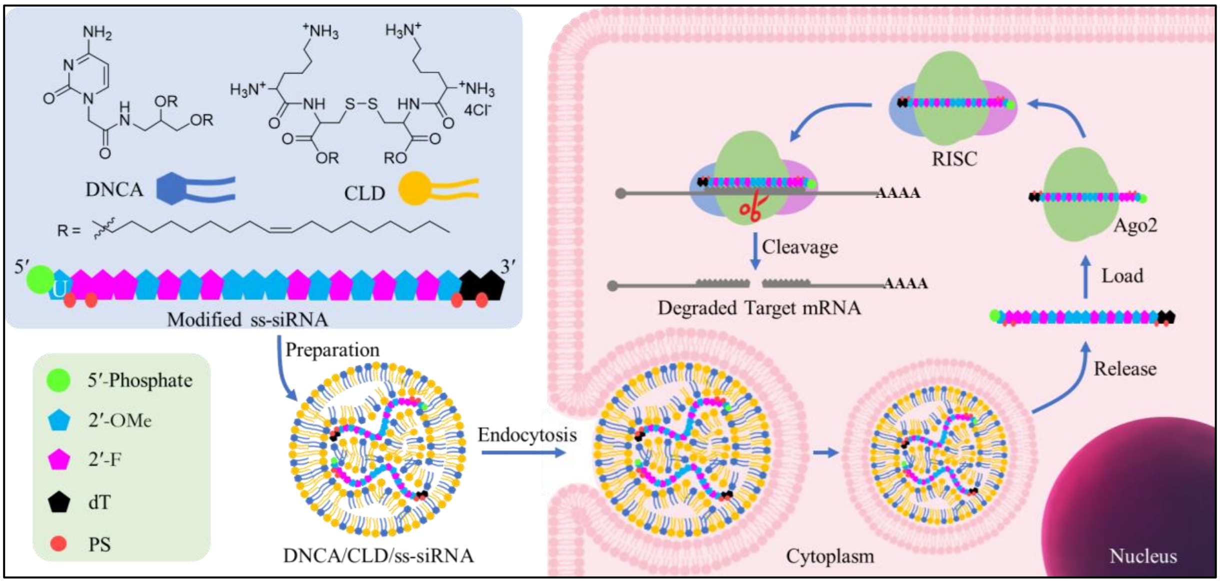

Outlined description: Modified ss-siRNA was delivered into the cells aiding with neutral cytidinyl/cationic lipids (DNCA/CLD), after release, ss-siRNA was loaded into Ago2 and form into RISC, activated RISC degraded the target mRNA efficiently.

[†]These authors contributed equally to this paper.
[*]Correspondence author. Zhenjun Yang: Tel. & Fax: +86-10-82802503. E-mail: yangzj@bjmu.edu.cn.
